# The Future of TDR: The Need to Adapt to a Changing Global Health Environment

**DOI:** 10.1371/journal.pntd.0000310

**Published:** 2008-11-25

**Authors:** Daniel J. Carucci, Michael Gottlieb

**Affiliations:** 1 United Nations Foundation, Washington, D.C., United States of America; 2 Foundation for the National Institutes of Health, Bethesda, Maryland, United States of America; *PLoS Neglected Tropical Diseases*, United States of America

In this issue of *PLoS Neglected Tropical Diseases*, Abdallah Daar and colleagues describe the fourth independent external analysis and evaluation of the Special Programme for Research and Training in Tropical Diseases (TDR) and summarize the findings resulting from the review [1]. The panel responsible for the review and report, which was chaired by Daar, recommends several critical areas of refocus and reorganization and calls for additional funding of TDR. In making its recommendations, the panel considers the changes that have taken place over the last decade. The most notable change was the unprecedented increase in funding, external to TDR, for research and control of tropical diseases and the accompanying establishment of product development partnerships (PDPs).

The panel contends that TDR should increase its focus on research capacity strengthening, concentrate on neglected populations over neglected diseases, and strengthen its role in transdisciplinary research, particularly by augmenting its capacity in social sciences. In spite of TDR's clear successes and accomplishments since its inception, the panel concludes that TDR has not kept pace with a dynamic and changing world of global health research. The panel also concludes that TDR has not established itself as a credible partner with other leading funders, and is in danger of being marginalized to the point of ineffectiveness in addressing potentially critical gaps in tropical disease research. The review suggests that in its current form, TDR is overly bureaucratic and poorly aligned with the World Health Organization (WHO), that it has insufficient funds and flexibility to carry out its mandate, and that it is not readily able to adapt to the rapidly evolving and dynamic global health landscape.

In order to ensure that TDR takes its proper role in supporting research and development (R&D) and training and to capitalize on the tremendous support the organization has among a broad constituency, especially among scientists in disease-endemic countries, the report recommends that TDR focus its efforts in four specific areas: (1) stewardship, (2) expanded interventional research, (3) research capacity strengthening, and (4) R&D for physical products that are not otherwise supported. In doing so, and with the recognized need for substantial increases in funding, TDR must dramatically rethink its objectives and organization. It should also focus efforts on improved relationships with its sponsoring organizations and forge new interactions with organizations, especially public–private partnerships, with complementary interests in R&D and capacity building.

In their response to Daar and colleagues' external review, Robert Ridley (the Director of TDR) and colleagues acknowledge many of the shortcomings identified in the review, and say that TDR has committed to a series of steps to improve and reorganize based on the evaluation's recommendations [2]. These steps include a revised strategic focus on knowledge management, an increased capacity building effort, and an enhanced focus on neglected areas such as some aspects of translational research. The external review contributed to TDR's new Ten Year Strategy and Business Plan approved by TDR's Joint Coordinating Board and endorsed by WHO.

To implement the new strategy, Ridley et al. describe the development of “business lines” such as “BL3: Lead Discovery for Drugs” or “BL7: Accessible Quality Assured Diagnostics.” These business lines are supported by expert scientific advisory committees, and by necessity the business lines can be started or stopped depending on circumstances and the needs of the stakeholder community. It is envisioned that this business line model will provide a better means for TDR to make decisions and to respond to changing priorities. These business lines cover the full product development pathway from basic research through product development to research for access to interventions. They are viewed as a critical mechanism to decentralize TDR into discrete functional units (rather than to decentralize administratively) that is more responsive to changing environments and to the priorities of TDR's stakeholders. Ridley et al. correctly point out that TDR has played a key role in the establishment of PDPs, such as the Medicines for Malaria Venture. However, TDR's influence in this area has been overshadowed by others, and consequently TDR proposes to shift its focus from supporting PDPs to other areas where it can have greater influence and impact.

TDR has made major contributions to the lives of those in the developing world and has supported scores of students and scientists from disease-endemic countries. With funding from UNICEF, the United Nations Development Programme, the World Bank, and WHO, TDR is considered the developing world's research arm. It is uniquely positioned to identify key areas in tropical disease research and training that are not being met by other funding agencies, and to seek to fill those gaps either through direct funding or by creating partnerships that leverage other investments. In recent years, there has been an increased recognition that the scientists, public health workers, and policy makers in disease-endemic countries should contribute to research priorities. TDR is in a position to make these voices heard in setting the priorities. Although TDR has had successes assisting in the establishment of PDPs, it has not fared so well in partnering with other donor agencies, and as such may be moving toward marginalization at a time when it could be playing a critical role in filling important research, training, and implementation gaps.

The establishment of business lines is intended to functionally decentralize TDR and make it more responsive to its stakeholders. It will be important, however, to see how the business line concept is functionalized, how lines are initiated and terminated, how funding priorities are made across business lines, and what metrics will be established to measure the progress toward each business line's objectives. Are the business lines repackaged programs, or are they truly a new means of setting and managing research priorities?

TDR's strengths and successes will need to be better marketed, and its credibility as a major player in the donor community will have to be strengthened. It will need to actively seek to establish better and more effective means to partner with others. It will need to identify those areas where it is uniquely positioned to make a significant impact and determine the measure of that success, both in terms of its longstanding and broad reach in partnership with the developing world and its ability to extend the impact of the efforts of other donors.

The global health community and external landscape have changed dramatically since the establishment of TDR in 1978. TDR has made positive contributions to these changes. One thing is certain: the landscape will continue to change as research provides new opportunities to extend and improve the lives of those in the developing world; as innovations transition from bench to bedside; as new and exciting partners recognize the importance of contributing to the gargantuan effort needed; as new scientists from the developed and developing world enter into the scene; and as the priorities of the developing world change. TDR must be given the strength, flexibility, and resources to play a major role in extending progress in tropical disease research and training.

## References

[1] Daar AS, Whyte SR, Abdullah MS, Ching-Li H, Hoffman SL (2008). TDR thirty years on: taking stock and envisioning the future for the Special Programme for Research and Training in Tropical Diseases.. PLoS Negl Trop Dis.

[2] Ridley RG, Ndumbe P, Korte R (2008). Two years after the Fourth External Review: TDR moves forward with a new vision and strategy.. PLoS Negl Trop Dis.

